# Length of Individual Apnea Events Is Increased by Supine Position and Modulated by Severity of Obstructive Sleep Apnea

**DOI:** 10.1155/2016/9645347

**Published:** 2016-03-09

**Authors:** Timo Leppänen, Juha Töyräs, Anu Muraja-Murro, Salla Kupari, Pekka Tiihonen, Esa Mervaala, Antti Kulkas

**Affiliations:** ^1^Department of Clinical Neurophysiology, Kuopio University Hospital, Puijonlaaksontie 2, 70210 Kuopio, Finland; ^2^Department of Clinical Neurophysiology, Seinäjoki Central Hospital, Hanneksenrinne 7, 60220 Seinäjoki, Finland; ^3^Department of Applied Physics, University of Eastern Finland, Yliopistonranta 1, 70211 Kuopio, Finland; ^4^Institute of Clinical Medicine, Faculty of Health Sciences, University of Eastern Finland, Yliopistonranta 1, 70211 Kuopio, Finland

## Abstract

Positional obstructive sleep apnea (OSA) is common among OSA patients. In severe OSA, the obstruction events are longer in supine compared to nonsupine positions. Corresponding scientific information on mild and moderate OSA is lacking. We studied whether individual obstruction and desaturation event severity is increased in supine position in all OSA severity categories and whether the severity of individual events is linked to OSA severity categories. Polygraphic recordings of 2026 patients were retrospectively analyzed. The individual apnea, and hypopnea durations and desaturation event depth, duration, and area of 526 included patients were compared between supine and nonsupine positions in different OSA severity categories. Apnea events were 6.3%, 12.5%, and 11.1% longer (*p* < 0.001) in supine compared to nonsupine position in mild, moderate, and severe OSA categories, respectively. In moderate and severe OSA categories desaturation areas were 5.7% and 25.5% larger (*p* < 0.001) in supine position. In both positions the individual event severity was elevated along increasing OSA severity category (*p* < 0.05). Supine position elevates apnea duration in all and desaturation area in moderate and severe OSA severity categories. This might be more hazardous for supine OSA patients and therefore, estimation of clinical severity of OSA should incorporate also information about individual event characteristics besides AHI.

## 1. Introduction

The classification and severity of obstructive sleep apnea (OSA) are traditionally based on apnea-hypopnea index (AHI). AHI < 5 events/h is judged as normal, 5 ≤ AHI < 15 events/h as mild OSA, 15 ≤ AHI < 30 events/h as moderate OSA, and AHI ≥ 30 events/h as severe OSA [1]. However, AHI takes into account only the number of the breathing cessation events per hour of sleep neglecting the duration and morphology of the individual respiratory events. The severity of individual respiratory events can be estimated based on the duration of apnea and hypopnea events and the morphology (i.e., depth, duration, and area) of desaturation events. In general, longer apnea and hypopnea and longer and deeper desaturation events may be considered to be more severe than the shorter and shallower ones. Oksenberg et al. (2000) reported that, in patients with severe OSA, the apnea events are longer and the difference between the minimum and maximum values of arterial oxyhemoglobin saturation events is greater in the supine than in the lateral position. However, there is no published information available about the differences in durations of individual apnea and hypopnea events or morphology of individual desaturation events between the supine and nonsupine positions in mild and moderate OSA. It is common belief that individual apnea and hypopnea events are more severe (longer) in the supine compared to the nonsupine positions, despite the OSA severity category. However, there is no solid scientific evidence on this. It has been shown that AHI is higher in supine position despite the sleep stage and regardless of whether or not OSA is more severe in NREM (nonrapid eye movement) compared to REM (rapid eye movement) sleep [2, 3]. Apnea events have been found to be longer in supine position [4] but also in REM compared to NREM sleep [3–5]. However, there are no differences in durations of apnea or hypopnea events in supine compared to nonsupine position during REM sleep [2]. In these studies, separation between different OSA severity categories was not done or patient pools contained only specific patients (e.g., obese individuals) and therefore, these findings may not be generalized to all OSA patients.

In the present study, we explored the possible differences in the length of the apnea and hypopnea events and in the morphology of desaturation events between the supine and nonsupine positions in all OSA severity categories (mild/moderate/severe). We hypothesize that the durations of individual respiratory events and morphology of desaturation events differ between sleeping positions and that they are modulated by the severity of OSA. The systematic review of literature was made in PubMed in December 2015. Based on the literature review there are no previous studies on the effect of sleeping position on individual characteristics (i.e., duration and morphology) of obstruction events in different OSA severity categories.

## 2. Patients and Methods

In this study, we reanalyzed retrospectively the ambulatory polygraphic recordings of 2026 adult patients with clinically suspected OSA, studied at the Department of Clinical Neurophysiology at Kuopio University Hospital during the years 1992–2003. The study was given a positive statement by the Research Ethics Committee of the Hospital District of Northern Savo, Kuopio, Finland (decision numbers 127/2004 and 24/2013). The polygraphic recordings were conducted with a custom-made four-channel ambulatory device (Neurotech OY, Kortejoki, Finland) designed by one of the authors (PT). The device recorded airflow with a triple-head thermistor (Philips type 2322 626 22103, Philips, Eindhoven, Netherlands), abdominal respiratory movements with a piezoelectric sensor placed above the lowest rib on the right side, and the blood oxygen saturation by a finger pulse oximeter (Minolta Pulsox-7, Konica Minolta, Tokyo, Japan). The four main positions (right, left, supine, and prone) were detected using a sensor based on gravitational tilt switches. In the sensor, two gravitationally sensitive mercury tilt switches were fixed at a 90° angle towards each other and at a 45° angle to the supine position plane [6, 7]. In addition, all recordings were reanalyzed using the standard American Academy of Sleep Medicine (AASM) respiratory rules [8]. Hypopnea events were scored if amplitude of the thermistor signal did drop ≥30% from the reference level for at least 10 seconds causing ≥4% drop of desaturation signal (rule 4A) [8]. Apnea events were scored if the thermistor amplitude did drop ≥90% from the reference level for at least 10 seconds [8]. These rules were used for scoring of apnea and hypopnea events according to the AASM recommendations and clinical practice of Kuopio University Hospital at the time of the analysis.

From the original group of 2026 patients, 11 patients were excluded due to insufficient information on sleeping position. Further, only those patients who had at least 60 minutes of supine and at least 60 minutes of nonsupine recording time during the night were included in the subsequent analyses (552 patients excluded). It was also required that patients have had apnea, hypopnea, and desaturation events in both supine and nonsupine positions (937 patients were excluded as they did not meet this criterion). The purposes of these requirements were to ensure that the patients had slept sufficiently in both positions and that the comparison between respiratory events occurring in supine and nonsupine positions would be possible. It must be acknowledged that this selection excluded patients whose apneic events are completely resolved in nonsupine positions. Eventually 526 patients (466 males and 60 females) were included in the final study. The demographics of the included patients are shown in Table 1.

**Figure 1:**
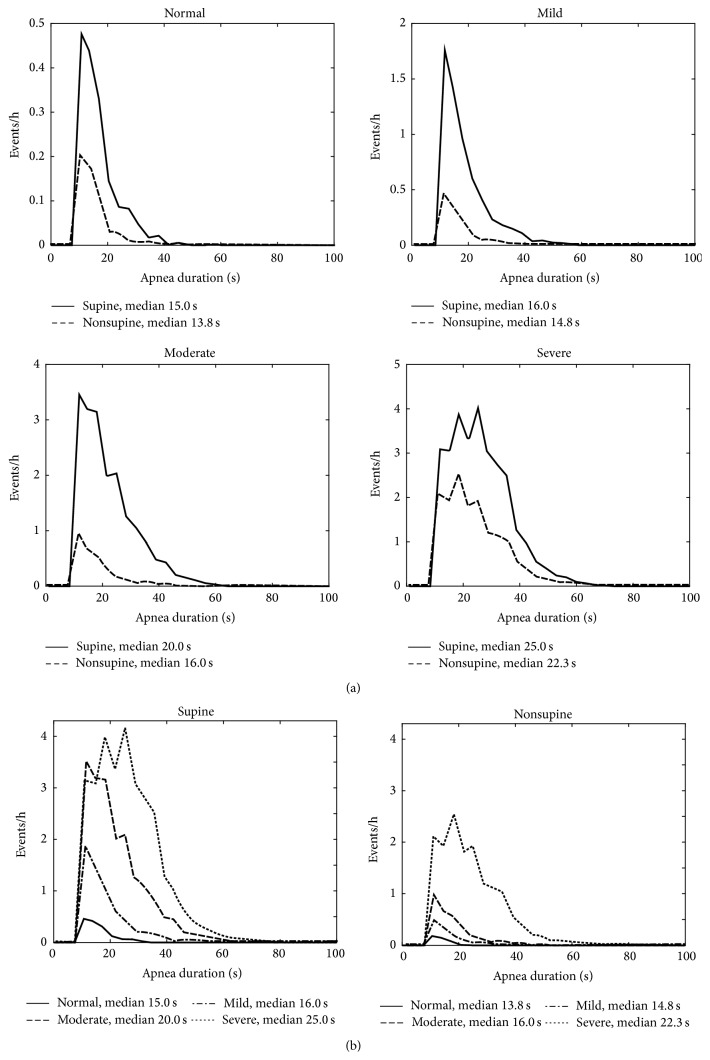
(a) Positional sleeping time adjusted distributions of the durations of individual apnea events in supine and nonsupine positions in different OSA severity categories and (b) distributions in nonsupine and supine positions as a function of OSA severity. In all OSA severity categories the apnea events are longer in supine position than in the nonsupine position. Based on mixed model analysis, the differences are statistically significant in all OSA severity categories. Also the median durations of apnea events increase along increasing OSA severity in both supine and nonsupine positions.

**Figure 2:**
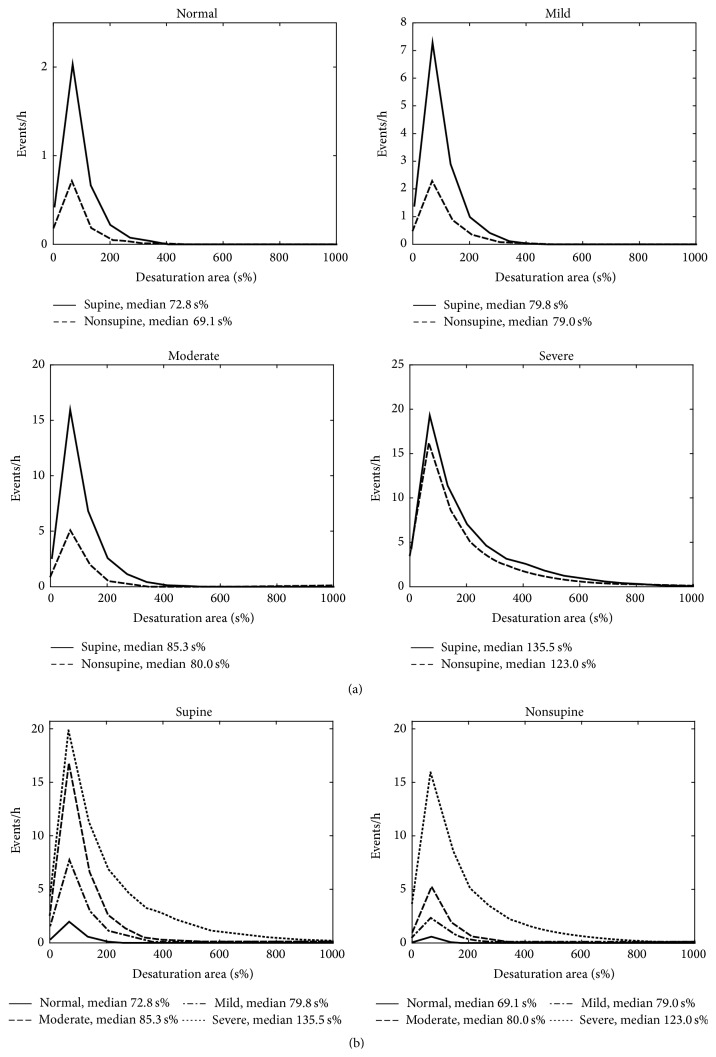
(a) Positional sleeping time adjusted distributions of the areas of individual desaturation events in supine and nonsupine positions in different OSA severity categories and (b) distributions in nonsupine and supine positions as a function of OSA severity. In all OSA severity categories the areas of the desaturation events are larger in the supine position compared to the nonsupine position. Based on mixed model analysis, the differences are statistically significant in the moderate and severe OSA severity categories. The median desaturation areas increase along increasing OSA severity in both supine and nonsupine positions.

**Figure 3:**
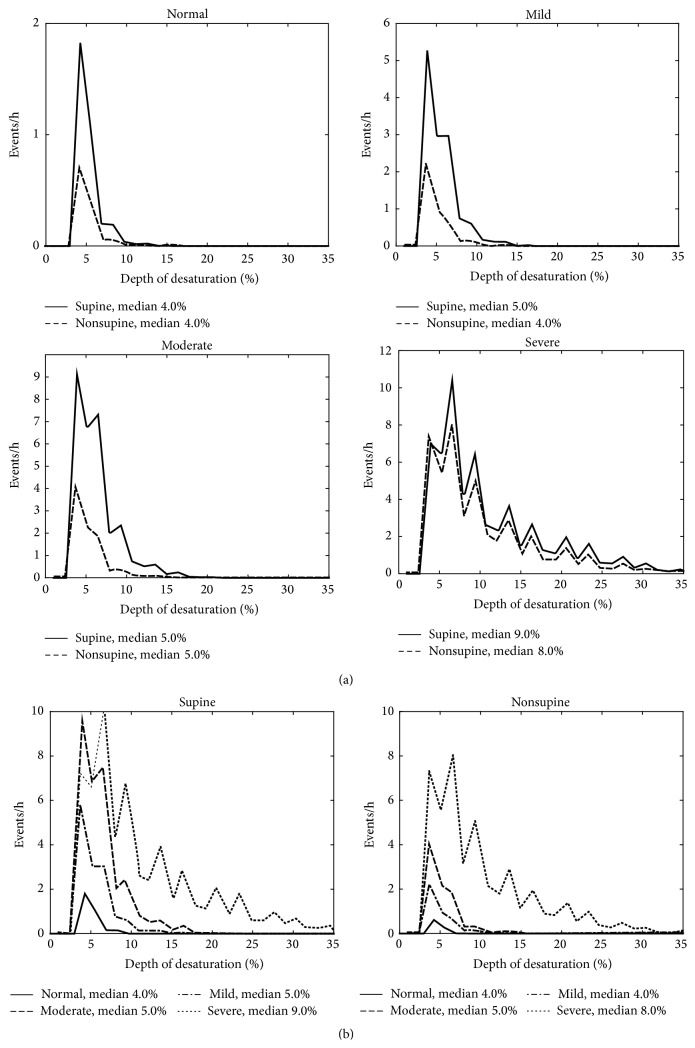
(a) Positional sleeping time adjusted distributions of the depths of individual desaturation events in supine and nonsupine positions in different OSA severity categories and (b) distributions in nonsupine and supine positions as a function of OSA severity. In mild and severe OSA categories the median depths of the desaturation events are greater in the supine position than in the nonsupine position. Based on mixed model analysis, the differences are statistically significant in all OSA severity categories excluding normal category.

**Figure 4:**
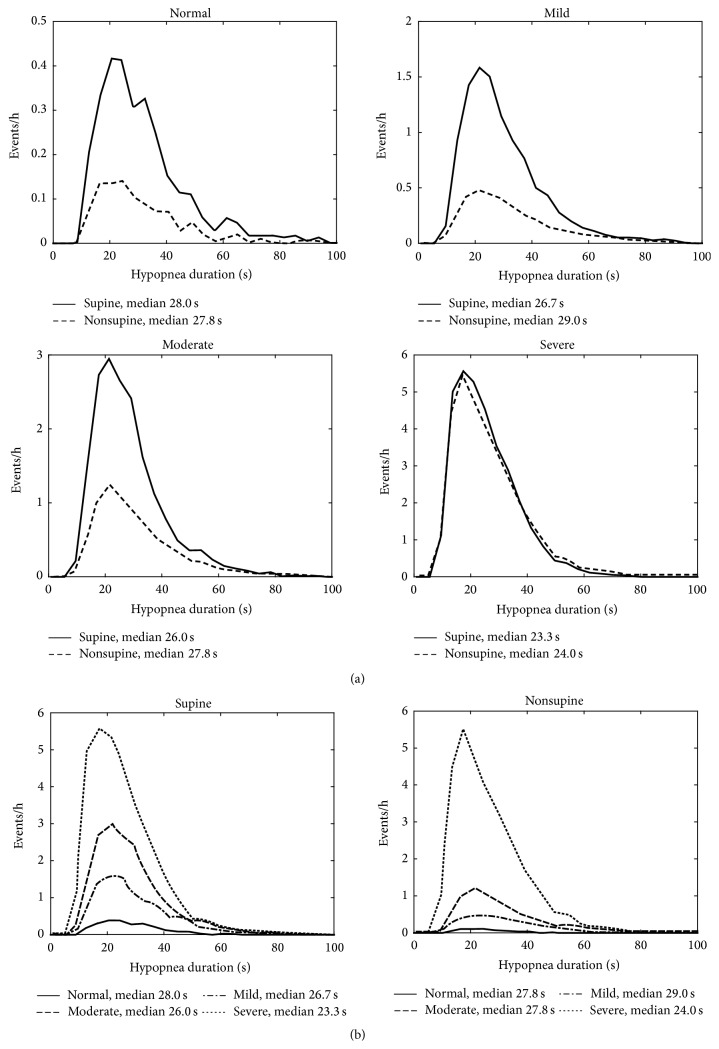
(a) Positional sleeping time adjusted distributions of the durations of individual hypopnea events in supine and nonsupine positions in different OSA severity categories and (b) distributions in nonsupine and supine positions as a function of OSA severity. In every OSA severity category, excluding normal category, the hypopnea events were slightly longer in nonsupine position than in supine position. Based on the mixed model analysis, the differences are statistically significant in all OSA severity categories excluding the normal category.

**Figure 5:**
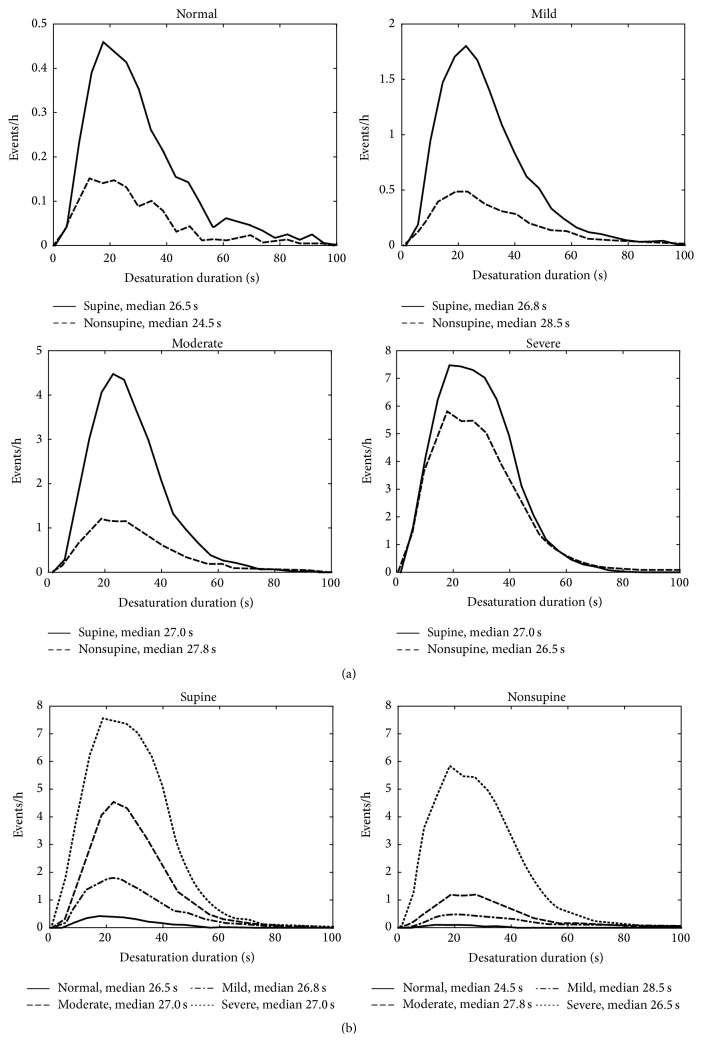
(a) Positional sleeping time adjusted distributions of the durations of individual desaturation events in supine and nonsupine positions in different OSA severity categories and (b) distributions in nonsupine and supine positions as a function of OSA severity. In mild and moderate OSA categories the median durations of the desaturation events are longer in nonsupine position than in supine position. The differences in the distributions of desaturation event durations are statistically significant in all OSA severity categories based on mixed model analysis.

The patients were classified into the clinical OSA severity categories (normal/mild/moderate/severe) based on total AHI. The median values of apnea, hypopnea, and desaturation event durations, desaturation event area, and maximum desaturation depth value as well as proportion of apnea events were calculated for each patient in both supine and nonsupine positions and comparison between these values in different positions was made (Table 2). Also AHI, AI (apnea index), HI (hypopnea index), and ODI (oxygen desaturation index) were calculated and compared between supine and nonsupine positions (Table 1). Furthermore, the individual event data (i.e., apnea and hypopnea event durations and desaturation event duration, area, and depth) of all patients were collected in two separate pools in each OSA severity category. The first pool included data recorded in the supine and the second pool data recorded in the nonsupine position. Individual event data recorded in supine and nonsupine positions were further normalized by dividing the number of events by the total recording time in supine and nonsupine position, respectively. The normalized distributions of the individual event data for different positions (supine and nonsupine) were computed (Figures 1–5) by dividing the *x*-axis into 50 equal wide bins between the 0 and maximum value of the parameter in question (duration, area, or depth).

Desaturation depth was determined as the difference between the minimum and maximum values within desaturation event. The starting point of a desaturation area event was set to the first baseline point before the onset of the drop and the end was set to the point before the oxygen saturation values started to rise again. Interval between these two points (i.e., duration of desaturation event in question) was divided into multiple bars. The width of the bars was defined by sampling interval and the height as a difference between the saturation value and baseline saturation value. Desaturation area was defined as a sum of areas of these bars.

The statistical significance of the differences in the median values of respiratory and desaturation events occurring in supine and nonsupine positions in different OSA severity categories was investigated using Wilcoxon signed rank test. The statistical significance of differences between the OSA severity categories was determined with Kruskal-Wallis test. Mixed model analysis was performed to assess differences in individual event distributions between the positions as it takes into account the correlation structure of data due to multiple measurements per individual. The individual apnea, hypopnea, and desaturation durations as well as desaturation depths and areas were further standardized and the residuals of the corresponding models were made normally distributed to facilitate interpretation of the results. In the mixed model, these individual event data were used as the dependent variables. Sleeping position (supine or nonsupine) and OSA severity categories (normal/mild/moderate/severe) were used as fixed effects and the patient was used as a random effect in the mixed model analysis. The normality of model residuals was judged by visual inspection of their histograms. Statistical analyses were made using SPSS (version 19.0, SPSS Inc., Chicago, IL, USA) software and the limit of the statistical significance was set to *p* < 0.05.

## 3. Results

In the patients with mild, moderate, or severe OSA the median values of apnea event durations and the proportion of apnea events were higher (*p* < 0.05) in supine position compared to nonsupine position (Table 2). Similarly, the median values of depths of desaturation events were significantly (*p* < 0.001) higher in supine compared to nonsupine position (Table 2). In addition, the median values of the desaturation event areas were greater (*p* ≤ 0.001) in supine position in patients with moderate or severe OSA (Table 2). On the contrary, in patients with moderate OSA, hypopnea events were longer in the nonsupine position (*p* = 0.001). There was no statistically significant position dependent variation in the median values of durations of the desaturation events in any OSA severity category (Table 2).

In general, the median apnea durations and desaturation areas increased in both supine and nonsupine position towards more severe OSA (Table 2). The proportions of apnea events in supine position (31.2%, 43.5%, and 43.5%) were greater (*p* < 0.05) compared to those in nonsupine position (25.0%, 23.1%, and 24.1%) in mild, moderate, and severe OSA categories, respectively.

In the mixed model analysis, considering all respiratory events obtained from all patients, statistically significant (*p* < 0.05) differences in values of apnea event durations between supine and nonsupine positions were seen in all OSA severity categories (Figure 1). Statistical significant (*p* < 0.05) differences in desaturation event areas between supine and nonsupine positions were also found in moderate and severe OSA categories (Figure 2). Desaturation event areas were larger and apnea event durations longer in supine position compared to the nonsupine position (Figures 1 and 2). Based on visual inspection of individual event distributions the durations of apnea events occurring in supine position were distributed over the wider range while in nonsupine position the durations of apnea events were distributed towards the shorter ones (Figure 1). The depths of desaturation events showed a rather similar behavior (Figure 3). In contrast, the areas of desaturation events were distributed almost over the same range in both positions (Figure 2) and the durations of hypopnea and desaturation events almost over the same range regardless of the position or OSA severity category (Figures 4 and 5).

## 4. Discussion

The present study is based on retrospectively reanalyzed polygraphic recordings of 526 suspected OSA patients whose individual obstruction events were compared between supine and nonsupine positions in different OSA severity categories. The individual apnea events were found to be longer in patients with AHI ≥ 5 and desaturation areas greater in patients with AHI ≥ 15 in supine compared to nonsupine position. Also the durations of individual apnea events and areas of individual desaturation events elevated along increasing OSA severity category. In contrast, durations of desaturation or hypopnea events did not increase due to supine position or along increasing OSA severity category. It has been previously shown that AHI and AI are significantly increased in supine position compared to the nonsupine positions [3, 5, 9–12]. Also in the current study, AHI, AI, and ODI were significantly greater in supine position compared to the nonsupine position in all OSA severity categories (Table 1). Longer apnea events and longer and deeper desaturation events have been shown to have effect on the severity of OSA and promote severe OSA related health consequences [13]. As the number of events is greater in supine position compared to the nonsupine position and longer events are more harmful than shorter ones, even moderate increase in the durations of apneic events and in the desaturation areas especially in supine position, such as that seen in the current study, may lead to considerable increase in the total physiological stress and risk of severe health consequences related to all OSA severity categories. Therefore, the current findings further highlight the importance of respiratory events occurring in supine position when estimating the total clinical severity of OSA.

According to our best knowledge, the position dependency of the severity of individual obstruction events has been previously comprehensively investigated only in patients with severe OSA [14]. It has been shown that apnea events are longer in REM than NREM sleep [3–5] and there are no positional differences in apnea duration during REM sleep [2].

The number and duration of apnea events were greater in the supine compared to the nonsupine position in patients with OSA. Our results agree with previous studies in which severity of OSA, based on AI, was reported to increase due to supine position [9, 12] and apnea events were reported to be longer in supine position in patients with severe OSA [14]. As a new and important finding, we found that apnea events were statistically significantly (*p* < 0.001) longer in supine compared to nonsupine position also in the mild and moderate OSA severity categories (Figure 1 and Table 2). Interestingly, Oksenberg et al. (2010) reported an opposing result: apnea durations did not differ statistically significantly between sleeping positions. This might be due to the fact that Oksenberg et al. (2010) did not make separation between different OSA severity categories. Presently, the median apnea event duration was found to increase with increasing OSA severity in both positions (Figure 1 and Table 2). It has been shown that a single night sleep recording may underestimate AHI due to abnormally short sleep time in supine position during the night of the recording [10] and that the severity of the individual obstruction events is connected to increased risk of mortality and cardiovascular morbidity [13]. Since the time spent in supine position varies from night to night, apnea events appear to be longer and more frequent in supine position and the risk ratios of mortality and cardiovascular morbidity are elevated in the supine dominant OSA patients [15]; these should be taken into account when estimating the overall severity of positional OSA. In addition to AHI, one option might be to evaluate the overall severity of OSA based on adjusted-AHI parameter, incorporating the number, duration, and morphology of the individual obstruction events [16], in different positions.

From the clinical perspective, the information of individual event characteristics may provide supplementary information to conventional AHI especially in cases where a patient with mild or moderate OSA has serious and persistent OSA related symptoms. We found that there are great differences in median durations of apnea, hypopnea, and desaturation events between individual patients albeit the patients are classified into the same OSA severity category (Table 2). In addition, the median depths and areas of desaturation events differ between individuals within the same OSA severity category, too (Table 2). As longer apnea and hypopnea events together with longer and deeper desaturation events are, most likely, more hazardous than shorter and shallower ones and may lead to increased physiological stress experienced by the patient, AHI might not be sufficient to estimate overall severity of OSA. Therefore, more detailed information about durational and morphological characteristics of individual respiratory events should be used in classification and estimation of clinical severity of OSA.

No statistically significant difference in the patient specific individual hypopnea durations between supine and nonsupine positions was seen except in the patients with moderate OSA whose hypopnea events were found to be longer in the nonsupine position (Table 2). The durations of hypopnea events were distributed almost over the same range in all OSA severity categories despite the sleeping position (Figure 4). It has been previously shown that the relative number of apnea events is significantly elevated with increasing severity of OSA [17]. It might be speculated that instead of lengthening the hypopnea events will turn into apnea events as disease progresses but also while sleeping in supine position. This assumption is supported by the present findings that the proportion of apnea events was increased statistically significantly (*p* < 0.05) in the supine position in the patients with AHI ≥ 5 (Table 2). However, it is acknowledged that further research is needed to explain the somewhat unexpected findings in the hypopnea durations. It has been shown that the cross-sectional area of upper airways (UA) is reduced in OSA patients [18] and that supine position causes further narrowing of the pharynx [19]. The gravity causes anatomical changes in the UA during the supine position resulting in higher UA resistance leading to breathing difficulties during sleep increasing the probability of UA obstruction [20]. These anatomical changes might explain the elevated proportion of apnea events in supine position. As the cross-sectional area of the pharynx is smaller in supine position the probability that UA is completely blocked might be higher.

It has been shown that in patients with severe OSA the individual desaturation events are deeper in supine position [14]. Our results are in line with this, as the depths of the desaturation events were found to be greater (*p* < 0.001) in supine compared to the nonsupine position in all OSA severity categories except in normal category (Figure 3 and Table 2). As there was no statistically significant difference in desaturation event duration between supine and nonsupine positions (Table 2) the significant increase in the desaturation event areas in moderate and severe OSA categories is probably due to increase in desaturation depth. In addition, the median desaturation event areas increased along increasing OSA severity in both positions (Figure 2 and Table 2). This can affect the overall severity of OSA as deeper desaturation events are reported to increase physiological stress (e.g., sympathetic activity and oxidative stress) [21]. As ODI takes into account only the number of desaturation events but depth alters between sleeping positions and is modulated by the severity of OSA, these should be taken into account when the severity of OSA is estimated based on ODI. As previously introduced novel* desaturation severity* parameter [7, 22] incorporates also the durations and depths of the desaturation events, it might be a better predictor of severity of OSA than conventional ODI.

In the present study, ambulatory polygraphic recordings were reanalyzed retrospectively. These recordings did not include electroencephalography (EEG) and therefore hypopnea events followed by arousal could not be detected and more sensitive AASM respiratory rule 4B could not be used [8]. The lack of EEG causes uncertainty in detection of total time in sleep. In the present study, the start and end points of the analyzed period were selected based on polygraphy signals. This might have slightly influenced the determined parameters as they are normalized with the sleep time. Despite these shortcomings ambulatory polygraphic devices not including EEG are currently considered to be sufficient for diagnostics of OSA [23]. In addition, differences in durations of apnea and hypopnea events and durations and morphology of desaturation events between REM and NREM sleep in different sleeping positions and in different OSA severity categories could not be examined due to the lack of EEG recording. This issue clearly warrants being further investigated using polysomnographic recordings including EEG. However, all the recordings were performed with identical devices and therefore we believe that these technical shortcomings do not undermine the results significantly. In addition, the patient population of this study contained both male (*n* = 466) and female (*n* = 60) patients. The smaller number of female patients might be due to the fact that the prevalence of OSA is generally lower among female compared to male patients [24]. Due to the limited number of female patients, the data was not analyzed separately by gender. It is acknowledged that due to this it is not possible to make broad generalizations of the results on both genders.

## 5. Conclusion

In conclusion, the number and severity of the individual apnea events and desaturation events were higher in the supine compared to the nonsupine position in all OSA severity categories. Further, the duration of individual apnea events and area of the individual desaturation events increased with increasing severity of OSA. As the apnea and hypopnea event durations and desaturation areas are linked to morbidity and mortality of OSA patients and alter depending on the position and the severity category of OSA, the current findings highlight the importance of analyzing individual event characteristics besides AHI when estimating the overall severity of OSA.

## Figures and Tables

**Table 1:** The patient demographic data, median (range), in different OSA severity categories formed based on total AHI. Total analyzed time is denoted as TAT (min).

	Normal		Mild		Moderate		Severe	
	Supine position	Nonsupine position	Supine position	Nonsupine position	Supine position	Nonsupine position	Supine position	Nonsupine position
Patients (*n* (females))	90 (12)		158 (23)		117 (13)		161 (12)	
Age (years)	45 (26–67)		50 (28–75)		52 (33–81)		53 (21–81)	
BMI (kg/m^2^)^#^	26.6 (21.1–41.8)		28.1 (19.9–44.6)		30.1 (21.2–53.7)		34.9 (21.1–55.8)	
AHI total (events/h)^#^	2.6 (0.6–4.9)		9.4 (5.0–14.9)		21.3 (15.1–29.5)		50.2 (30.0–148.4)	
AHI (events/h)	4.9 (0.7–24.6)	1.4 (0.3–5.1)^*∗*^	16.6 (3.6–65.7)	3.9 (0.5–20.4)^*∗*^	39.6 (2.7–77.9)	8.7 (0.4–30.8)^*∗*^	63.9 (16.4–179.6)	42.2 (0.9–125.5)^*∗*^
AI total (events/h)^#^	0.7 (0.2–4.1)		2.4 (0.2–12.6)		8.9 (0.4–24.6)		19.1 (0.3–81.6)	
AI (events/h)	1.2 (0.2–22.7)	0.3 (0.1–3.1)^*∗*^	4.7 (0.2–45.1)	0.7 (0.1–9.2)^*∗*^	17.0 (0.4–67.3)	1.7 (0.2–14.8)^*∗*^	27.6 (0.3–86.0)	7.4 (0.1–73.0)^*∗*^
HI total (events/h)^#^	1.7 (0.2–4.6)		5.8 (0.4–13.9)		12.4 (1.0–25.2)		29.5 (1.6–115.2)	
HI (events/h)	2.6 (0.2–16.6)	0.7 (0.1–3.7)^*∗*^	9.2 (0.7–54.9)	2.6 (0.1–20.1)^*∗*^	17.5 (1.3–72.3)	5.7 (0.2–30.3)^*∗*^	32.3 (0.4–167.7)	24.5 (0.4–102.5)^*∗*^
ODI total (events/h)^#^	2.1 (0.2–4.9)		7.3 (0.7–14.6)		18.0 (4.4–29.2)		47.6 (15.0–149.0)	
ODI (events/h)	3.3 (0.2–17.5)	0.9 (0.1–3.5)^*∗*^	13.7 (0.7–65.7)	3.3 (0.2–20.4)^*∗*^	31.3 (2.7–77.9)	6.8 (0.2–29.7)^*∗*^	59.6 (14.2–187.3)	41.6 (0.4–120.8)^*∗*^
TAT (min)	159 (61–385)	294 (103–496)^*∗*^	147 (60–409)	275 (62–466)^*∗*^	163 (60–438)	259 (78–394)^*∗*^	192 (60–446)	225 (63–445)^*∗*^

^*∗*^ *p* < 0.05, significant difference between events occurring in supine and nonsupine positions, Wilcoxon signed rank test.

^#^ *p* < 0.05, significant dependence on the severity of the OSA, Kruskal-Wallis test.

**Table 2:** The individual event data, median (range). In mild, moderate, and severe OSA categories median values of apnea event durations, proportions of apnea events (of all respiratory events), and the maximum desaturation depths were significantly greater in supine position. Desaturation areas were significantly larger in supine position in patients with moderate and severe OSA. Hypopnea events were statistically significantly longer in nonsupine position in moderate OSA category.

	Normal			Mild			Moderate			Severe		
	Supine position	Nonsupine position	*p* value	Supine position	Nonsupine position	*p* value	Supine position	Nonsupine position	*p* value	Supine position	Nonsupine position	*p* value
Patients (*n*)	90			158			117			161		
Hypopnea duration (s)	29.0 (12.5–68.0)	27.8 (15.0–127.7)	0.499	27.0 (13.8–56.3)	27.4 (11.0–74.5)	0.257	26.5 (16.0–42.0)	27.3 (13.0–61.0)	**0.001**	25.0 (13.0–54.0)	25.0 (12.3–63.0)	0.221
Apnea duration (s)	14.6 (10.1–28.5)	13.9 (10.0–43.9)	0.144	15.1 (10.1–55.6)	14.2 (10.0–51.0)	**<0.001**	18.0 (10.7–44.0)	16.0 (10.8–92.5)	**<0.001**	22.0 (10.5–52.2)	19.8 (11.0–64.3)	**<0.001**
Proportion of apnea events (%)	33.3 (3.8–92.0)	34.8 (5.3–80.0)	0.859	31.2 (1.0–97.8)	25.0 (1.2–94.7)	**0.022**	43.5 (1.3–97.7)	23.1 (1.3–91.2)	**<0.001**	43.5 (0.8–99.5)	24.1 (0.4–99.0)	**<0.001**
Desaturation duration (s)	25.9 (8.8–118.5)	23.3 (6.5–81.6)	0.220	26.6 (11.0–54.9)	27.4 (5.5–72.5)	0.324	27.0 (13.3–43.8)	27.5 (9.8–64.1)	0.570	27.3 (6.8–59.0)	27.5 (8.9–70.4)	0.347
Desaturation area (s%)	70.8 (23.5–337.0)	67.6 (15.0–245.3)	0.064	75.6 (29.5–240.0)	75.7 (24.5–187.1)	0.294	83.3 (39.4–345.5)	78.8 (24.0–184.8)	**0.001**	127.3 (35.3–795.3)	101.8 (22.9–807.5)	**<0.001**
Desaturation depth (%)	4 (4–18)	4 (4–12.5)	0.401	5 (4–13)	4 (4–8.5)	**<0.001**	5 (4–15)	4.5 (4–11.5)	**<0.001**	8 (4–36)	6 (4–33)	**<0.001**
Maximum desaturation depth (%)	7 (4–28)	6 (4–25)	**0.014**	10 (4–27)	8 (4–23)	**<0.001**	13 (5–33)	9 (4–40)	**<0.001**	23 (9–70)	20 (4–62)	**<0.001**
Number of hypopnea events	746	444		4696	2635		6281	3820		18033	19663	
Number of apnea events	420	246		2735	915		6461	1530		16014	9441	
Number of desaturation events	899	512		6039	3033		10731	4537		32512	27918	

The patients were divided into OSA severity categories based on the total AHI. The statistical significance of differences between events occurring in supine and nonsupine positions was evaluated using the Wilcoxon signed rank test. *p* < 0.05 was considered as the limit for statistical significance.
